# Limits of the social-benefit motive among high-risk patients: a field experiment on influenza vaccination behaviour

**DOI:** 10.1186/s12889-020-8246-3

**Published:** 2020-02-17

**Authors:** Ozan Isler, Burcu Isler, Orestis Kopsacheilis, Eamonn Ferguson

**Affiliations:** 1 Centre for Behavioural Economics, Society and Technology (BEST), School of Economics and Finance, Queensland University of Technology (QUT), Brisbane, 4000 Australia; 2 Centre for Decision Research and Experimental Economics (CeDEx), University of Nottingham, Nottingham, UK; 3 Infectious Diseases and Clinical Microbiology Department, Sisli Hamidiye Etfal Training and Research Hospital, Istanbul, Turkey; 4 Centre for Clinical Research, The University of Queensland, Herston, Brisbane, 4029 Australia; 5 School of Economics, University of Nottingham, Nottingham, NG7 2RD UK; 6 School of Psychology, University of Nottingham, Nottingham, NG7 2RD UK

**Keywords:** Vaccination, Influenza, Field experiment, Social benefit, Risk group, Risk perceptions, Framing, Nudge

## Abstract

**Background:**

Influenza vaccine uptake remains low worldwide, inflicting substantial costs to public health. Messages promoting social welfare have been shown to increase vaccination intentions, and it has been recommended that health professionals communicate the socially beneficial aspects of vaccination. We provide the first test whether this *prosocial vaccination hypothesis* applies to actual vaccination behaviour of high-risk patients.

**Methods:**

In a field experiment at a tertiary care public hospital in Istanbul, Turkey, we compare the effects of two motivational messages for promoting vaccination. Using a between-subjects single-blind experimental design patients were randomly assigned to frames emphasizing the vaccine’s benefits to self (*n* = 125) or social benefits (*n* = 119). Free influenza vaccination was offered to each patient.

**Results:**

Among 222 patients who were not vaccinated for the season prior to the study (72% medically assessed to be at high risk), 42% in the self-benefit frame chose to receive a vaccination compared with 34% in the social-benefits frame, but the difference was not statistically significant (aOR = 1.63, 95% CI 0.90 to 2.95, *p* = 0.108). Reasons for vaccination focused primarily on self-benefit (67%) rather than social-benefit (5%). Exploratory analysis showed that the effect of messages depended on patient perception of risk group membership (aOR_High_ / aOR_Low_ = 5.59, 95% CI 1.30 to 24.05, *p* = 0.021). In particular, emphasis on self-benefit was more influential among patients who perceived themselves to be in the risk group (aOR = 6.22, 95% CI 1.69 to 22.88, *p* = 0.006).

**Conclusions:**

In contrast to the literature observing intentions of low-risk populations, we found no evidence that social-benefit motivates actual vaccination behaviour among a high-risk patient population. Instead, those who self-categorize as being in the high risk group are more motivated by the self-benefit message. Our results suggest that a stratified approach can improve coverage: even if an emphasis on social-benefit could be effective among low-risk groups, an emphasis on self-benefit holds more promise for increasing vaccination in medical organizational settings where high-risk groups are prevalent.

**Trial registration:**

ClinicalTrials.gov NCT04230343 Retrospectively registered on the 13th January 2020.

## Background

Influenza poses a serious threat to human health, annually resulting in 250,000 to 500,000 deaths worldwide [1]. Even though the influenza vaccine is widely available and affordable, its global uptake remains low [2]. Meanwhile, the negative impact of influenza is expected to grow due to rapid increases in high-risk populations such as the elderly [3]. As long as herd immunity remains a distant goal, vaccination of high-risk populations will remain a public health priority [4], despite vaccination’s limited effectiveness in such populations [5]. Furthermore, even with accrued knowledge of the social and psychological correlates of vaccination [3, 6–8], causal pathways that can be exploited to increase uptake remain poorly understood, rendering the behavioural impact of public policies ambiguous. In particular, experimental investigations of vaccination behaviour among high-risk patient groups are rare. Our study hence focuses on behavioural interventions to motivate vaccination among those at heightened risk of harm from influenza infection.

A promising candidate for promoting vaccination is the idea of harnessing prosocial motives (e.g., care for family, friends and community) by highlighting the social benefits of vaccination [9]. Various studies report evidence that messages about the social benefits of vaccination strengthen the *intentions* to vaccinate [10–12]. Based on these findings, it was recently recommended that professionals can increase vaccine uptake by emphasizing its social benefits [13]. We refer to this view as the *prosocial vaccination hypothesis*. Given accumulated evidence for the prevalence of prosocial motives in other domains [14, 15], the idea is promising. However, evidence for the prosocial vaccination hypothesis remains limited for two reasons.

First, the general applicability of the prosocial vaccination hypothesis is not well-established. In particular, the effects of health behaviour interventions will likely depend on psychological differences [7, 16, 17] such as risk perceptions [18–21]. Supporting this view, prosocial sensitivities have been found to decrease with disease risk [22], and the effect of motivational messages have been found to depend on the relative prevalence of high and low risk groups [23].

Second, most experiments investigating social benefit as a vaccination motive measured intentions among low-risk populations in artificial settings [9, 11, 12, 22, 23]. However, intentions do not always predict behaviour well [24–26]. In particular, hypothetical scenarios may enhance a preference to appear altruistic [27, 28] and thus limit generalizability of previous evidence [29, 30]. The only two field experiments testing the hypothesis found no advantage of prosocial messages in promoting vaccination, yet they studied the behaviour of health-care professionals and not the more prevalent patient groups [31, 32]. It hence remains unknown whether social welfare motivates actual vaccination uptake of high-risk groups in real-world settings [9].

To overcome these two limitations, we conducted the first field experiment in a hospital setting that observed the causal effects of prosocial messages on the vaccination behaviour of high-risk patients in a natural environment. We used two motivational frame manipulations, one emphasizing the self-benefit and the other emphasizing the social-benefit of vaccination. The two frames used in our experiment were based on actual vaccine promotion pamphlets employed by the UK National Health Service. The prosocial vaccination hypothesis predicts that emphasizing the social benefits of vaccination will increase uptake compared to an emphasis only on benefits to self.

Perceived risk has been shown to be a more powerful predictor of vaccination than objective risk [33]. We compare objective (medically diagnosed) and subjective (patient perceived) measures of *risk group* status, indicating high or low risk of severe harm due to influenza related illnesses [8], and explore whether they moderate the effect of prosocial messages on vaccination behaviour. We provide self-reported reasons for vaccine acceptance and refusal as well as a rationale why social-benefit messages may be counterproductive for those who perceive themselves as being in the risk group for influenza complications.

## Methods

### Participants

**Fig. 1:**
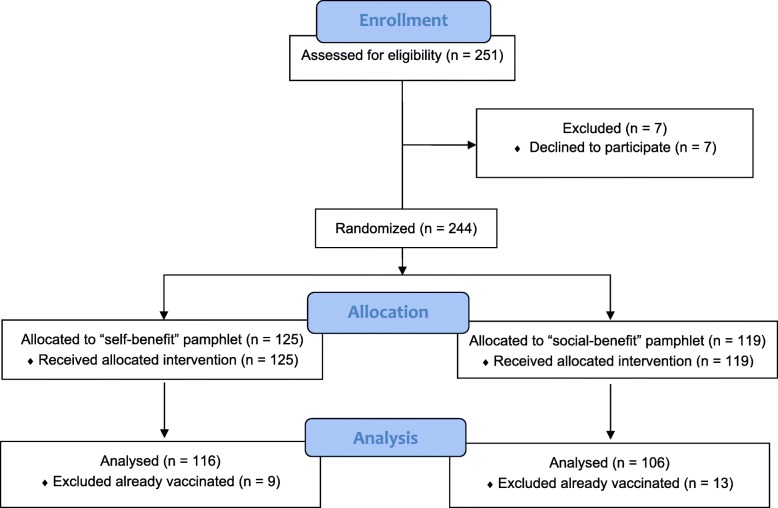
Study flow

Participants were recruited at a tertiary care public hospital in Istanbul from November 2016 to March 2017. Two hundred and fifty-one adult patients were approached during the twenty-week study period and 244 of them (97%) agreed to participate. One hundred and sixteen patients were female (48%), the median age was 60 and only 54 patients had education levels higher than primary school (22%). Patients were recruited from the Internal Medicine (41%), Neurology (28%), Infectious Diseases (23%), Physical Medicine (7%) and Dermatology (1%) wards. Eighty-six participants were diagnosed with an infectious disease at admission to the hospital (35%). The two most common infection diagnoses were pneumonia (42%) and urinary tract infections (17%). Other diagnoses of infections included skin and soft tissue infections, intra-abdominal infections, viral hepatitis, central nervous system infections, HIV infection, etc. Patients were randomly assigned to either the self-benefit or the social-benefit message. Twenty-two of the 244 patients (9%) reported that they were already vaccinated for the season (see Fig. 1 & Table 1).

**Table 1:** Characteristics of participants in analysis across the treatment arms (*n* = 222)

	Social-benefit	Self-benefit	*p*-value*
Age, median years (mean ± SD)	59 (57 ± 17)	60 (56 ± 18)	0.68
Gender			
Female	47 (44)	59 (56)	0.33
Male	59 (51)	57 (49)	
Diagnosis at admission			
Infectious disease	32 (41)	47 (59)	0.11
Other diagnosis	74 (52)	69 (48)	
Ward			
Infectious Diseases	22 (42)	31 (58)	0.57
Internal Medicine	46 (53)	41 (47)	
Neurology	31 (48)	34 (52)	
Other	7 (41)	10 (59)	
Education			
Less than high school degree	81 (47)	93 (53)	0.50
High school degree or above	25 (52)	23 (48)	
Objective risk group (medical assessment)			
High	77 (48)	82 (52)	0.75
Low	29 (46)	34 (54)	
Subjective risk group (patient perception)			
High	26 (51)	25 (49)	0.60
Low	80 (47)	91 (53)	

Note. Number of persons (%), unless otherwise indicated. * *t*-test for age, χ^2^ otherwise

### Power calculations

Because there is no previous comparison of the effects of self- and social-benefit on actual patient vaccination behaviour, we assumed a medium effect size (aOR = 3.0 or a Cohen’s *d* of 0.60) [34, 35]. This value is smaller than the previously found effect (*d* = 0.74) of communicating social benefit when individual benefit was already conveyed and when vaccination was immediately accessible [10], as was the case in our study. We planned for a two-tailed logistic model (α = 0.05 & 1-β = 0.80) and aimed to recruit a total of 159 objectively high-risk patients, not yet vaccinated for the flu season, for the two treatment groups. Until we reached our high-risk patient target, we also recruited low-risk patients, who were less prevalent among hospital in-patients. We consequently recruited 159 objectively assessed high-risk and 63 low-risk patients who were not yet vaccinated for the flu season as well as 22 patients who were already vaccinated for the season.

### Procedure

**Fig. 2:**
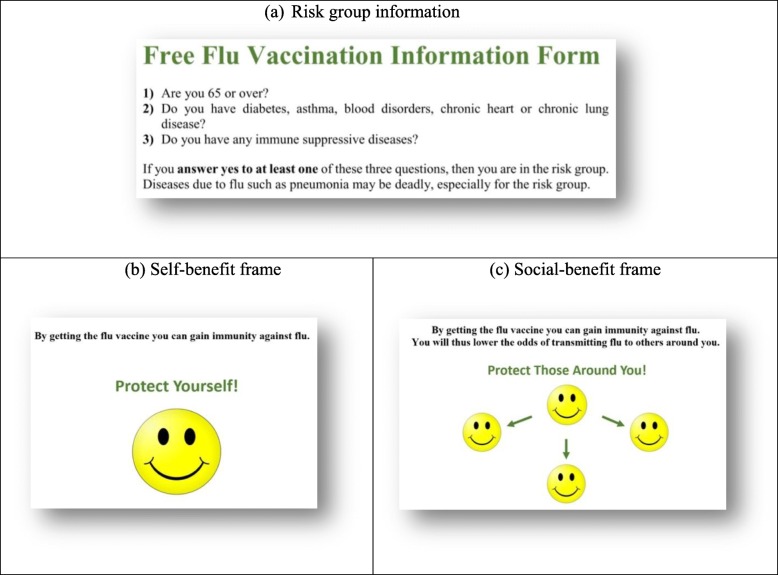
Experimental Treatments

One of the researchers, an infectious disease physician, recruited in-patients on the day of their discharge. By selecting the next pamphlet on the top of a previously shuffled stack, patients were randomly assigned to one of two treatment groups, including either the self-benefit (51%) or the social-benefit message treatment (49%). The pamphlets were modelled after actual vaccine promotion messages used by NHS Wales.^1^ Each treatment consisted of short text and an abstract figure, facilitating the transparency of the message to a relatively old and poorly educated participant pool (see Fig. 2). The top halves of the two pamphlets were the same and described the official criteria for qualifying to be in the risk group (Fig. 2a). At the bottom half, the text in the self-benefit treatment indicated that one can gain immunity against influenza by getting the vaccine (Fig. 2b), while the text in the social-benefit treatment in addition stated that gaining immunity would lower the chances of transmitting the disease to others (Fig. 2c). Prompts of “protect yourself” vs. “protect those around you” and corresponding emoticons were added to increase the salience and the clarity of each message [36–38]. Consistent with the pamphlets used by NHS Wales, no explanation of herd immunity was provided, and both pamphlets listed the objective risk group criteria and stated that influenza can have serious complications especially for someone in the risk group.

The pamphlets were given after eliciting written informed consent and between two questionnaires that sought basic demographic information as well as knowledge, experience, attitudes and perceptions regarding influenza and its vaccine (see Additional file 1 for the questionnaires). Care was taken to limit any further verbal interaction between the researcher and the patients. We allowed only two types of patient requests to change the course of the interaction. First, the researcher uniformly recommended the vaccine only if asked for her advice (30%). Second, the researcher read out the pamphlet when help was requested (55%) or otherwise left the room for 5 min. Assistance for reading was sought primarily due to old age and illiteracy. In these cases, information flow on the pamphlets was used as the script. The binary variables of asking for recommendation (Self-benefit: 29%; Social-benefit: 30%) and asking for help with reading (Self-benefit: 57%; Social-benefit: 52%) did not systematically differ between the two treatment conditions.

Upon the researcher’s return to the room and prior to the second questionnaire, the decision of whether or not to get the freely provided flu vaccine was elicited. Participants who decided to receive the free vaccine were vaccinated on site at the end of the study, which comprised the primary outcome measure.

After the elicitation of vaccination decision, as part of the second questionnaire (see Additional file 1: Table S1), reasons for accepting or refusing vaccination as well as perceptions for being in the high risk-group (i.e., subjective risk) were measured by asking “Why do you [not] want to get vaccinated?” (Q2.1) and “Are you in the risk group for flu?” (Q2.2). In contrast, patients were categorized as at high objective risk through medical assessment based on the criteria set out by the Turkish Ministry of Health at the time of the study and listed on the pamphlets for defining high-risk of mortality due to influenza-related illnesses: elderly (≥65), people with various chronic illnesses (e.g., diabetes, asthma). Those who did not belong to any of these groups formed the low objective risk group.

As a secondary outcome measure, all participants were finally given a free vaccination ticket that could be used by patient’s family and friends within 2 weeks. This was intended to explore whether social networks can boost vaccination beyond the hospital setting. Since information on the tickets was provided after elicitation of vaccination decisions (as well as after the questionnaires), this additional feature could not influence the primary outcome measure.

### Analysis

Twenty-two of the 244 patients (9%) have indicated in the questionnaires that they were already vaccinated for the season. Hence, we restricted our analysis of treatment effects to the 222 patients who were not yet vaccinated (74% medically assessed to be at high risk) and who randomly received either the self-benefit (52%) or the social-benefit (48%) treatment.

We recorded the two types of endogenous variation in interventions—patients asking for recommendation and patients asking for the pamphlet to be read out loud for them—and we control for them as covariates in our analysis. First, we estimated a simple logistic regression to find the effect of the prosocial message on vaccination (Model A). Next, we estimated the moderation of the treatment effect by objective risk in a logistic model that includes the experimental treatment, objective risk group status and their interaction (Model B). Using a similar interaction model, we then explored the moderation of the treatment effect by perceived risk-group membership (Model C). To ensure that any effect we observe is not due to the inclusion of the two aforementioned covariates [39], we ran these models twice, once with (the adjusted models) and a second time without the covariates (the unadjusted models).

Finally, we summarized reasons provided for vaccine acceptance and refusal, examined the overall impact of the intervention on vaccine uptake, and described the outcome of the free vaccine ticket program.

## Results

Among the 222 patients in the analysis, 159 were medically assessed to be in the high risk group (72%), whereas only 51 of 222 patients perceived themselves at high risk (23%). One hundred and twenty-eight of 222 patients (58%) miscategorized their risk group status. Among these patients with inaccurate risk perceptions, 118 underestimated their risk (92%) as compared to 10 patients who overestimated their risk (8%).

We find no evidence that an emphasis on the vaccination’s social-benefits increases vaccination as compared to an emphasis on its self-benefit. In fact, as shown in Model A in Table 2, vaccination uptake in the self-benefit treatment was 8 percentage points higher than in the social-benefit treatment. However, this difference was not statistically significant (aOR = 1.63, 95% CI 0.90 to 2.95, *p* = 0.108). As shown in Model B on Table 2, the difference in vaccination rates between the two treatment groups also did not depend on whether patients were medically assessed to be at high or low risk of influenza-related complications (aOR_High_ / aOR_Low_ = 0.88, 95% CI 0.22 to 3.55, *p* = 0.856).

**Table 2:** Effect of frames on vaccination and its moderation by objective and subjective risk group

Determinant	Vaccinated patients / Patients in category (%)	Adjusted		Unadjusted	
		Subgroup	Interaction	Subgroup	Interaction
		aOR [95% CI]	aOR_High_/aOR_Low_ [95% CI]	OR [95% CI]	OR_High_/OR_Low_ [95% CI]
Model A: Overall Effects of Frames					
Social-benefit	36/106 (34.0)	1		1	
Self-benefit	49/116 (42.2)	1.63 [0.90–2.95]		1.42 [0.82–2.46]	
Model B: Moderation of framing effects by objective risk (medical assessment)					
Low Risk Group	15/63 (23.8)				
Social-benefit	6/29 (20.7)	1	1	1	1
Self-benefit	9/34 (26.5)	1.82 [0.53–6.17]		1.38 [0.42–4.49]	
High Risk Group	70/159 (44.0)				
Social-benefit	30/77 (39.0)	1	0.88 [0.22–3.55]	1	1.08 [0.28–4.13]
Self-benefit	40/82 (48.8)	1.60 [0.80–3.17]		1.49 [0.79–2.81]	
Model C: Moderation of framing effects by subjective risk (patient perception)					
Low Risk Group	62/171 (36.3)				
Social-benefit	29/80 (36.3)	1	1	1	1
Self-benefit	33/91 (36.3)	1.11 [0.56–2.20]		1.00 [0.53–1.87]	
High Risk Group	23/51 (45.1)				
Social-benefit	7/26 (26.9)	1	**5.59 [1.30–24.05]**	1	**4.82 [1.25–18.56]**
Self-benefit	16/25 (64.0)	**6.22 [1.69–22.88]**		**4.83 [1.46–15.92]**	

Note. Table presents vaccination rates and describes corresponding logistic model estimates for three models. Model A describes the overall effect of message frame treatment on vaccination. Model B describes the interaction between objective risk and treatment, whereas Model C describes the interaction between subjective risk and treatment. Adjusted estimates include two covariates: doctor’s recommendation and reading of the pamphlet. Unadjusted estimates provide consistent results. Robust SE. *p* < 0.05 in bold

As shown in Model C on Table 2, exploratory analysis of perceived risk group status showed a significant moderation of the treatment effect among the overall sample (aOR_High_ / aOR_Low_ = 5.59, 95% CI 1.30 to 24.05, *p* = 0.021). Specifically, the self-benefit message significantly increased vaccination more than the social-benefit message among those who perceived themselves as being in the high risk group (aOR = 6.22, 95% CI 1.69 to 22.88, *p* = 0.006) while it had no additional effect on vaccination among those who perceived themselves as being in the low risk group (aOR = 1.11, 95% CI 0.56 to 2.20, *p* = 0.758). As seen in the unadjusted estimates columns on Table 2, these results are robust to the exclusion of the two covariates [39].

Among 72 patients who sought and received the doctor’s recommendation, 47 chose to receive vaccination (65.3%), in comparison to 38 of 150 (25.3%) patients who did not seek recommendation. This covariate was significant in all three models (Model A: aOR = 5.52, 95% CI 2.94 to 10.35, *p* < 0.001). Help with reading of the pamphlet was sought by 117 patients, 51 of whom vaccinated (43.6%). Among the 105 patients who did not seek such help, 34 vaccinated (32.4%). This covariate was not significant in any of the three models (Model A: aOR = 1.30, 95% CI 0.71 to 2.38, *p* = 0.394).

Patients (*n* = 222) were asked to provide reasons for their vaccination decisions, and answers were categorized as belonging to one of five acceptance or one of five refusal reason types (See Table 3). Two coders (OI & BI) independently assigned each answer to one reason type (Cohen’s kappa: 0.811 for vaccinated & 0.766 for refused). Given the high inter-rater reliability, any discrepancies in ratings were resolved by mutual agreement. Among those who decided to receive the vaccine as part of the study, the overwhelming majority cited “self-protection” as the reason (67%), whereas only four patients referred to protection of others (5%). Among those who decided not to receive the vaccine, responses that reflected confidence in one’s current health status such as “I am healthy”, “I don’t need vaccines”, and “I never catch the flu” were categorized as “self-confident”. The most common reasons for rejection were found to be “self-confidence” (26%), “current health conditions” such as receiving other treatments (26%), “vaccine mistrust” (15%) and “lack of experience or knowledge regarding the vaccine” (14%). Although “self-protection” was a more prevalent reason in the self-benefit (61%) than in the social-benefit treatment (39%), no statistically significant difference due to experimental manipulations was observed for any category in either acceptance or refusal reasons (Fisher’s exact tests: *p* = 0.423 and *p* = 0.738 respectively).

**Table 3:** Reasons for vaccine acceptance & refusal

Acceptance (*n* = 85)		Refusal (*n* = 137)	
Self-protection	57 (67.1%)	Self-confidence	36 (26.3%)
Recommendation	7 (8.2%)	Current-conditions	36 (26.3%)
Others’-protection*	4 (4.7%)	Vaccine mistrust	21 (15.3%)
Being in risk group	3 (3.5%)	Inexperience	19 (13.9%)
Other or no reason	14 (16.5%)	Other or no reason	25 (18.3%)

* Including “protection of self and others”

As a consequence of our intervention, 85 of 222 (38%) patients who were not yet vaccinated for the flu season chose to receive vaccination. Including the 22 participants who were already vaccinated at the start of the study, rate of vaccination by the end of the study was 44%. These values indicate substantial improvements over previous year rate of 16% vaccination among all 244 study participants (based on questionnaire item Q1.7, see SI) as well as over the 2006 vaccination rates of 6–19% among high-risk groups in Turkey [40].

*None* of the 244 free vaccination tickets were brought back to the hospital to receive free vaccination. Though we have no way of knowing, we conjecture that patients either failed to pass the tickets on to others or when they did, they failed to persuade others to vaccinate. Either of these cases can be interpreted as evidence against strong prosocial motives in vaccination among a high-risk group.

## Discussion

### Principle findings

This field experiment provides the first behavioural test of the prosocial vaccination hypothesis among a predominantly high-risk patient population. This hypothesis builds on tests of vaccination intentions in hypothetical scenarios [10–12], as well as on domain-general evidence that people care about the well-being of others [14, 15]. However, evidence suggests that an emphasis on social-benefits may not be as effective in a high-risk group. Indeed, direct self-benefit motives are often relatively stronger than social-benefit motives, especially when people feel personally at risk [22, 23]. Moreover, no previous study has provided decisive evidence from the field that prosocial messages increase vaccination coverage [9], and it has been suggested that an emphasis on self-protection may be more successful in increasing vaccination than an emphasis on its social benefits [32].

We compared the effectiveness of two alternative messages for motivating vaccination among a high-risk group facing real stakes. Failing to provide evidence for the prosocial vaccination hypothesis, the social-benefit message was not found to increase vaccination as compared to the self-benefit message. Although the difference was not statistically significant, average vaccination rate was 8 percentage points higher among those who received the self-benefit message than those who received the social-benefit message.

Furthermore, through our exploratory analysis, we found the self-benefit message to be significantly more effective among patients who *perceived* themselves to be in the high risk group. This is consistent with both empirical evidence that high-risk perceptions motivate preventive health behaviour such as vaccination [8, 19, 22, 23] and with theoretical evidence that self-focused message will be affectively more salient for those with higher perceptions of risk group membership [21]. Moreover, patients who accepted the vaccination provided reasons more frequently referring to the self-benefits of vaccination, while social-benefit motives were rarely mentioned. In contrast to perceptions of risk group membership, objective risk group membership did not moderate the effect of the messages on vaccine uptake. We invite future research to test our exploratory finding that high risk perceptions dampen the positive effects of social benefit messages.

A majority of our participants had risk misperceptions, most of whom had underestimated their risk. Risk group misperceptions have also been found to be relatively high in a random sample of the US population (34%), where most misperceptions were similarly due to underestimation (96%) [33]. These results suggest that understanding the determinants of risk misperceptions in general and risk underestimation in particular are likely to provide crucial insights into vaccination avoidance.

Our simple intervention achieved substantially higher influenza vaccine uptake as compared with high risk reference groups. Factors commonly present in hospitals such as the free provision of the vaccine, the low transaction costs of in-patients, the information provided in the pamphlets, and the presence of a medical authority are likely to have contributed to this outcome. Nevertheless, even when compared with the rate we achieved with our intervention, vaccination rates among high-risk groups in Western countries are substantially higher (65% in the US and 74% in the UK for ages 65 and older in 2011–2012) [3]. Prevalence of anti-vaccination attitudes, fear of side-effects and misplaced self-confidence of patients in Turkey may well explain this discrepancy [41–43].

### Limitations of the study

The difficulty of studying actual vaccination of natural risk groups in the field resulted in four limitations. First, we cannot identify the isolated effect of each message on vaccination uptake due to the lack of a “no intervention” control condition. Given our resources, we opted to maximize the number of observations in the two treatments. Second, description of the mechanism of herd immunity was not provided because our materials were adapted from pamphlets employed by an actual national health service, which refrained from a detailed explanation. Descriptions of herd-immunity have been shown to increase vaccination intentions [11], and it remains to be tested in promoting actual vaccination behavior among patients. Third, we were ethically obliged to present risk group criteria to both treatment groups, which may have resulted in the social-benefit message being mixed with information pertaining to self-benefit. Finally, as we explain in the next paragraph, the moderation of treatment effect by perceptions of risk group membership can be endogenous, although our analysis points to no such confounds.

We opted to elicit risk group perceptions after the vaccination decisions to avoid biasing of these decisions by the elicitation procedure. However, this design choice opens the measure to possible post hoc rationalizations, in particular, to patients’ inaccurate declarations that “they are not at risk” as justifications for their refusal to vaccinate [8]. We did not find evidence for such a confound: among those who were medically assessed to be at high risk, the accuracy of risk perceptions was not significantly different (χ^2^ test; *P* = 0.729) between those who refused (25%) and accepted vaccination (27%). Similarly, using post-treatment variables as covariates may bias model estimates if treatments affect these variables. Again, we find no influence of treatments on risk group perceptions (χ^2^ test; *P* = 0.598).

## Conclusions

Public authorities such as the CDC and the European Council prioritize the vaccination of those at most risk against influenza [3, 4]. Our field experiment showed that simple motivational interventions, when implemented in a real clinical setting, can effectively target and substantially increase the vaccination of such high priority groups. We also found that the prosocial vaccination hypothesis does not apply to high-risk groups. These findings do not necessarily contradict previous evidence supporting the hypothesis because social-protection afforded by vaccination can be more valuable than self-protection among the general public who are personally less at risk or among other cultures with stronger prosocial attitudes. Instead, in line with the impetus of the stratified medicine approach [44, 45], our results suggest a significant boundary condition on the prosocial vaccination hypothesis. We therefore suggest that heterogeneity of risk groups and their perceptions should be taken into account when formulating vaccine advocacy policies. In particular, professional communications that aim to promote vaccination among high risk groups may be more effective with an emphasis on vaccination’s self-benefit rather than its social benefits. More generally, our study highlights the importance of validating theoretical and laboratory findings in the field, and shows that a stratified approach to behavioural interventions can substantially improve the efficiency of public policies.

## Supplementary information

**Additional file 1: Table S1.** Responses to questionnaires and their relation to vaccination decisions.

Additional file 2: Dataset.

## Data Availability

All data generated or analysed during this study are included in Additional file 2.

## Abbreviations

aOR: Adjusted odds ratio
CDC: Centers for Disease Control and Prevention
CI: Confidence intervals
NHS: National Health Service
OR: Odds ratio
UK: United Kingdom

## Footnotes

1 The pamphlet is publicly available at https://www.nhsdirect.wales.nhs.uk/pdfs/Main%20Flu%20Poster%20WEB.pdf
