# Supplementary material for: Limits of the social-benefit motive among high-risk patients: a field experiment on influenza vaccination behaviour
Source: BMC Public Health. 2020 Feb 17;20:240. doi: 10.1186/s12889-020-8246-3 (PMC7027065; doi:10.1186/s12889-020-8246-3)
Supplement: Supplementary file 1 — Additional file 1: Table S1. Responses to questionnaires and their relation to vaccination decisions. [file 12889_2020_8246_MOESM1_ESM.docx]

**Supplementary Information**

**Limits of the social-benefit motive among high-risk patients: A field experiment on influenza vaccination behaviour**

Ozan Isler, Burcu Isler, Orestis Kopsacheilis, Eamonn Ferguson
